# Debunking health myths on the internet: the persuasive effect of (visual) online communication

**DOI:** 10.1007/s10389-022-01694-3

**Published:** 2022-01-19

**Authors:** Sabrina Heike Kessler, Eva Bachmann

**Affiliations:** Department of Communication and Media Research, University of Zurich, Andreasstrasse 15, 8057 Zurich, Switzerland

**Keywords:** Debunking strategies, Health myths, Correction of misinformation, Visual online health communication, Visualisation

## Abstract

**Aim:**

Developing evidence-based recommendations on how to debunk health-related misinformation and more specific health myths in (online) communication is important for individual health and the society. The present study investigated the effects of debunking/correction texts created according to the latest research findings with regard to four different health myths on recipients’ belief, behaviour and feelings regarding the myths. Further, the study investigated the effects of different visualisations (machine-technical created image, diagram, image of an expert, message without an image) in the debunking texts.

**Subject and methods:**

A representative sample of German Internet users (*N* = 700) participated in an anonymous online survey experiment with a 4 (myths) × 4 (picture) mixed study design.

**Results:**

The results show that receiving an online news article that refutes a widespread health myth with or without the use of an image can significantly change the attitudes of the recipients toward this myth. The most influential variable was the attributed credibility: the more credible a debunking text is for a recipient, the more corrective effectiveness it has. However, the corrective messages did not differ in their persuasive effects depending on the image types used.

**Conclusion:**

The results offer an optimistic outlook on the correction of health-related misinformation and especially health myths and insight into why and how people change their beliefs (or not) and how beliefs in health myths can be reduced. The findings can be used by journalists, scientists, doctors and many other actors for efficient (online) communication.

**Supplementary Information:**

The online version contains supplementary material available at 10.1007/s10389-022-01694-3.

## Introduction

The existence of health-related mis- and disinformation especially on the Internet has been evident not only during the novel coronavirus (SARS-oV-2) and COVID-19 (e.g., Arif et al. 2018; Pías-Peleteiro et al. 2013; Scullard et al. 2010), but for centuries. However, the enormous abundance of (uncertain) online information worldwide has led to what the WHO calls an ‘infodemic’ (World Health Organization [WHO] 2020), making it difficult for people to find evidence-based information and to distinguish correct from incorrect information. Whether the neologism *infodemic* is used properly here can be doubted (Simon and Camargo 2021), but what remains certain is that health-related misinformation has been shown to have a variety of negative consequences for individuals and society (Lewandowsky et al. 2021).

Developing evidence-based recommendations on how to adequately debunk health-related mis- and disinformation and myths in communication is important for not only individual health but also society as a whole (Cook et al. 2017; Swire and Ecker 2018). For example, the idea that the MMR vaccine causes autism is a myth whose spread poses a risk to society. It is still widespread on the Internet, although it has been repeatedly exposed as a myth in the media and debunked by strong scientific evidence (Scullard et al. 2010). The U.S. vaccination rate probably decreased significantly as a result of the spread of this myth (Poland and Spier 2010). The economic burden of measles outbreaks in the United States was estimated at several million dollars (Ortega-Sanchez et al. 2014; Smith et al. 2008); the number of measles cases has also increased in Germany in recent years (Robert Koch Institute 2018). Another example, denying the scientific consensus that HIV causes AIDS led to policies in South Africa between 2000 and 2005, which are estimated to have contributed to 330,000 excess deaths (Chigwedere et al. 2008).

Misunderstandings and inaccurate knowledge can occur because people in everyday life have limited time, cognitive resources, and/or motivation to understand complex scientific topics (Cook et al. 2017; Swire and Ecker 2018). Some people just believe what they have once heard or read from their parents and friends or online. However, regardless of how the misinformed beliefs were built, they are relatively stable in recipients’ cognitive/mental model, quite resistant to persuasive corrective messages and thus difficult to eliminate (Cook et al. 2017; Ecker et al. 2011; Lewandowsky et al. 2012; Swire and Ecker 2018). Corrective messages can even cause backfire effects, i.e. unintended effects when originally incorrect attitudes are further reinforced by receiving a correction message (Cook et al. 2017; Lewandowsky et al. 2012; Nyhan and Reifler 2014). Special debunking strategies are necessary to correct myths once established (Cook and Lewandowsky 2011).

Researchers have assembled a collection of recommended best-practice debunking strategies (e.g., Cook and Lewandowsky 2011; Dan 2021; Swire and Ecker 2018). Among other things they call for debunking texts that visually support the correction explanations (e.g., through graphics; Cook and Lewandowsky 2011; Dan 2021; Nyhan and Reifler 2019). A reason is the assumption that pictures can increase the perceived credibility of the core statement in a correction (Dan 2021).

The present study investigates the effects of debunking texts created according to the latest research for four different health myths on recipients’ belief, (future) behaviour and feelings regarding the health myths. The current state of research does not provide insights into how debunking strategies with different images can change the attitudes regarding health myths. The second aim of the study is to investigate the effect of different visualisations in the debunking texts. After discussing health myths and their distribution on the Internet, we review the research on debunking of misinformation and visual health communication and its effects. This forms the basis for the research questions and hypotheses. The research design and methodology of the online survey experiment and the results are presented next, followed by a discussion.

### Health myths and their distribution on the internet

Health myths can be defined as health-related statements that are generally disseminated, many people believe it and either are not supported by scientific evidence or have strong scientific evidence that speaks against rather than for them. Mostly they are pseudo-scientific explanations that may have intuitive appeal (Shmerling 2019). Originally, old health myths like “swimming after eating is dangerous” are typically provided by individually trustworthy sources, such as parents, grandparents and friends (Donovan and Thompson 2010; Northwell Health 2017), which is why they are credible and persistent. Health myths have also been reinforced and disseminated via the Internet (Cook 2019; Lewandowsky et al. 2012; Scullard et al. 2010). Many people (41%) who did not use the Internet to find health information say they usually ask their friends, relatives, or other people (Eurobarometer 2014). Health myths are mostly plausible, easily understood stories that sound like truth and wisdom. Many myths have arisen from outdated or misinterpreted scientific findings; others are couched in what seems like common sense or logic, such as that reading in the dark harms the eyes (Donovan and Thompson 2010; Vreeman and Carroll 2007) or alcohol enhances digestion (Heinrich et al. 2010). Therefore, myths are often based on a grain of truth in combination with misinterpretations, wishful thinking, or fears.

The actual spread of health myths on the Internet is still underresearched. The media conditions of the Internet, such as dynamics, multimedia, multimodality, reactivity and content personalization, make systematic data collection difficult, so the spread of health myths has not yet been analysed at all in the German-speaking world and only marginally in the international context. Primarily, the extent to which websites speak for or against vaccination myths was examined on a semantic-textual level. Pías-Peleteiro et al. (2013) assessed Spanish websites on the human papillomavirus (HPV) and concluded that 45.5% of all blogs and forums and 25% of all press sites analysed disseminated information on vaccination deterrence. Results from Scullard et al. (2010) and Madden et al. (2012) also showed that more or less false information is found depending on the health topic and that it varies by website type. For example, governmental, academic and non-profit websites can be trusted most when it comes to vaccination information, while sponsored sites are least trustworthy (Madden et al. 2012; Scullard et al. 2010). Since health information on the Internet is topic-dependent (Scullard et al. 2010) and location-dependent (Arif et al. 2018) dependent and these sources vary in their quality and accuracy, this also contributes to the dissemination of misinformation.

### Debunking of misinformation beliefs

Debunking is about changing the beliefs of recipients. This can be seen as a persuasive effect because persuasion in its broadest sense describes the process in which one actor attempts to change the beliefs of another social actor through the use of communication (Dillard 2010). Beliefs are defined by Wyer and Albarracín (2005) as “estimates of subjective probability which, in the case of propositions, are reflected in either (a) estimates of the likelihood that (b) expressions of confidence or certainty that the proposition is valid, or, in some cases, (c) agreement with the proposition” (p 277). They are thus to be regarded as subjective estimates of the probability that certain knowledge is true. A person’s beliefs can also be false, that is, they cannot correspond to the truth (Perloff 2017). Wyer and Albarracín (2005) describe beliefs as being based on verbal, emotional and visual information and able to be influenced cognitively as well as affectively and conatively. It can also be assumed that beliefs consist of a cognitive, affective and conative component (Kruglanski and Stroebe, 2008). These components can now potentially be influenced by communication. If this process is intentional, it can be *persuasion*. According to Perloff (2017), persuasion can be defined “as a symbolic process in which communicators try to convince other people to change their own attitudes or behaviours regarding an issue through the transmission of a message in an atmosphere of free choice” (p 22). The early definition by Bettinghaus and Cody (1987) also emphasises the persuasive effects that communication can have on beliefs, emotions and behaviour. Persuasion is “a conscious attempt by one individual to change the attitudes, beliefs, or behaviour of another individual or group of individuals through the transmission of some message” (Bettinghaus and Cody 1987, p 3).

Previous meta-analyses examining the effect of correction compared to uncorrected control conditions have shown that corrective messages can significantly reduce the belief in misinformation (Blank and Launay 2014; Chan et al. 2017; Walter and Murphy 2018). The exposure to a correction is better than not receiving a correction at all.

Researchers have assembled a collection of recommended best-practice debunking strategies (Cook and Lewandowsky 2011; Dan 2021; Ecker et al. 2015; Lewandowsky et al. 2012; Swire and Ecker 2018). For example, the debunking text should support credibility judgments (Swire and Ecker 2018). In mass communication, basing claims on evidence (e.g., study results or expert opinions), adequately referencing the evidence and presenting data in an comprehensible way will build credibility and thus contribute to a greater efficacy of the corrections (Gigerenzer et al. 2007). Furthermore, to avoid making people more familiar with misinformation (and thus risking a familiarity backfire effect), a debunking text should emphasise the intended facts rather than the myth (Cook and Lewandowsky 2011; Lewandowsky et al. 2012). A debunking text should also be simple, easily understandable and brief (clear language and graphs where appropriate; Cook and Lewandowsky 2011 and Lewandowsky et al. 2012). If the myth is simpler and more compelling than the debunking, it will be cognitively more attractive, which will risk an overkill backfire effect (Cook and Lewandowsky 2011; Lewandowsky et al. 2012). Further, an effective debunking text requires a factual replacement for the causal explanations initially supplied by the refuted misinformation (Cook and Lewandowsky 2011; Ecker et al. 2015). To effectively debunk misinformation, messages should provide a coherent and detailed explanation that enables recipients to update complete mental models and even describes why the misinformation was disseminated (Chan et al. 2017; Lewandowsky et al. 2012; Walter and Murphy 2018; Walter and Tukachinsky 2019). Last, graphical information is probably more effective than text in reducing misperceptions (Cook and Lewandowsky 2011; Dan 2021; Nyhan and Reifler 2019).

However, on average, correction does not entirely eliminate the effect of misinformation; there is a continued influence of misinformation (Walter and Tukachinsky 2019). The phenomenon of maintaining beliefs regarding explicitly contradictory evidence is also called “belief perseverance” (for an overview, see Anderson 2008). One reason for this is mental models, which appear to be the most consistently supported explanation for the (non)correction of misinformation (Walter and Tukachinsky 2019). It is assumed that health information forms mental models, which provide simple, causal explanations for facts and observations (Johnson and Seifert 1994). If these explanations, information, or whole health myths are now debunked, an unpleasant gap develops in that mental model. This is one reason why many people tend to believe in easily accessible myths that are incorrect but simple, coherent and complete (Cook and Lewandowsky 2011; Johnson and Seifert 1994; Lewandowsky et al. 2012). Representations of the valid and invalid information might also coexist side by side in memory and compete for activation (Swire and Ecker 2018). The theory of cognitive dissonance is based on the assumption that people tend to have cognitive consistency and desire consonant relationships between their cognitions. Cognitive dissonance is a state of mental imbalance resulting from inconsistent relationships between cognitions (Festinger 1957). Further, it is assumed that individuals not only attempt to reduce dissonance but also actively avoid information in which it is to be expected (Festinger 1957; see also confirmation bias, Pohl and Pohl 2004). In contrast, there are also processes in which people consciously reject corrective statements and thereby stabilise misinformation-motivated reasoning (Kraft et al. 2015). People are sometimes unwilling to accept new information, especially corrective information that contradicts their views (Cook et al. 2017; Nyhan and Reifler 2010). Although they know the scientific evidence, they refuse to accept it. It can therefore be noted that the rejection of scientific findings and the stability of misinformation are maintained not only by an uninformed population or the ever-increasing spread of misinformation but often by individually motivated characteristics and information processing (Cook et al. 2017).

Different factors can influence the effectiveness of debunking, such as individual predispositions, message factors, source factors etc. In terms of the nature of misinformation, research results show that the correction of real-world misinformation, which exist in real, as opposed to constructed misinformation, which are invented for a study, is more challenging (Walter and Murphy 2018). In terms of individual predispositions, studies show that a greater degree of scepticism can lead to a better refutation of misinformation (DiFonzo et al. 2016; Lewandowsky et al. 2012; Swire and Ecker 2018). The attributed credibility of a message and a source are regarded as decisive influencing variables (O’Keefe 2002; Pornpitakpan 2004). Using relevant evidence can increase a message’s credibility and persuasive effect. At the same time, corrections are less effective if the misinformation was attributed to a credible source (Walter and Tukachinsky 2019). Further, corrections are less effective if the misinformation was repeated multiple times prior to correction, or there was a time lag before the correction, both are mostly the case with health myths (Walter and Tukachinsky 2019). The longer a mental model of health myth is held, the more it becomes integrated into memory and difficult to eradicate (Ecker et al. 2015).

Value- and belief-incongruent news can often have backfire effects, which has already been proven for controversial topics, such as climate change (Hart and Nisbet 2012) or vaccine safety (Nyhan and Reifler 2014). These are unintended effects when originally incorrect attitudes are further reinforced by receiving a correction message (Cook et al. 2017; Nyhan and Reifler 2010,2014). The effect can be partially explained by the fact that people counterattack attitudinal mismatches/cognitive inconsistencies to strengthen their existing attitudes, for example, more attitudinally mismatched/cognitively inconsistent information is mentally activated than before the perception of a debunking message, which in turn leads people to report and have more extreme attitudes than before (Lodge and Taber 2000; Nyhan and Reifler 2010).

One focus of this study lies in professional debunking effects, according to the latest research on online debunking texts about health myths. Most previous studies on debunking of misinformation have focused mainly on the U.S. context and often used college students for convenience purposes as the meta-analysis of Walter and Murphy (2018) shows. However, nationality, education and age are relevant influence factors of debunking effectiveness (Walter and Murphy 2018). There is some weak evidence in the meta-analysis that corrections work better for student samples compared to nonstudent samples. The few empirical studies that highlight the intercultural facets of the debunking of misinformation support the possibility that corrective messages produce different outcomes in different societies (e.g., Cook and Lewandowsky 2016). This study aims to be representative in terms of age, gender and education in Germany.

- *Research Question 1: What is the persuasive effect of corrective messages on the Internet about health myths on recipients’ belief, (future) behaviour and feelings regarding the health myth?*
- *Hypothesis 1: The more credible a debunking text is perceived, the more corrective effectiveness a debunking text has.*

### Visual health communication and its effects

Health information in (online) newspapers is usually communicated textually and/or visually. In general, images in scientific and medical communication have a persuasive power, which can vary in their strength depending on the type of image (e.g., Arsenault et al. 2006; Kessler et al. 2016; McCabe and Castel 2008). Images that make evidence visible can be used as a persuasive tool because images generate more attention and are easier to process and remember than textual content and individuals consider most of what they can visually capture to be true (Holicki 1993). In online health communication, for example, images are often used as visual arguments for the evidence of research results or a proxy for the evidence of a fact. The evidence-giving illustrations range from Roentgen images and photographs to tables and diagrams to models and preparations (Arsenault et al. 2006). Diagrams present results in great detail, accurately and systematically and create more clarity and less opportunity for misinterpretation than purely linguistic mediation or numerical comparison do (Pluviano et al. 2017). The aim of diagrams is to illustrate structures, structural changes and connections and depict proportions (Isberner et al. 2013). Technical or machine-technical created images, such as MRI or Roentgen images, provide insights into areas that are inaccessible to the human senses (Lohoff 2008). In this context, they are regarded as measuring instruments as well as replacements for the perception of the human eye and thus also serve as empirical evidence (Lohoff 2008). A picture on which an expert is depicted is said to be less scientific and have less evidentiary power than diagrams or machine-technical created images (Kessler et al. 2016). However, experts on a picture convey authenticity and so also serve as credibility heuristic (Holicki 1993). The photograph of a single scientist can overcome a deference to science bias from a text-only weight-of-evidence article because it showcases an episodic frame in a visual format (Dixon et al. 2015). People then may use their recall of the exemplar when making judgments about what scientists in general believe (Dixon et al. 2015). All of these image types ultimately convey a wide variety of information and evidence and represent these in many ways.

Images can influence recipients’ attitudes and behaviour about a specific issue to a greater extent than a purely text-based message can (Holicki 1993; Kessler et al. 2016; Nyhan and Reifler 2019). In general, images enhance cognitive processing and generate more attention, which is why they are better remembered than textual content (Holicki 1993; Houts et al. 2006). The increased comprehensibility, cognitive processing and better memory lead to images also having a stronger influence on attitudes (Arsenault et al. 2006; Lohoff 2008). In general, images are learned, retained, understood and recognised more easily and quickly than words, since they are received in larger units than textual or verbal information, which recipients capture sequentially (Holicki 1993).

McCabe and Castel (2008) examined how the effects of different scientific image types differ from one another in a one-sided article, as did Kessler et al. (2016) in an article that focused on controversy. Both studies found that machine-technical created images have a stronger persuasive effect than the same message with diagram or image of an expert and texts with pictures had more persuasive effect than texts without pictures. Nyhan and Reifler (2019) analysed the effect of text and diagram on factual misperceptions regarding climate change. Their results show that the graphic information has a stronger impact on reducing misinformation than pure text. However, the text only depicted the information from the diagram and was not an original debunking text. Pandey et al. (2014) examined the persuasive influence of diagrams in comparison to tables on three different topics. The influence of prior attitudes becomes clear in this study; the graphics are more convincing when they reinforce recipients’ prior attitudes and when the initial attitude is not strongly polarised, charts seem to have a stronger effect than tables on persuasion likelihood and attitude change. Pluviano et al. (2017) compared the effect of text, tables and images of sick, unvaccinated children with the debunking of vaccine misinformation. The pictures had an influence, but no intervention strategies worked; the belief in vaccine myths and a desire not to vaccinate children increased over time (backfire effects).

How an image ultimately affects the recipients, however, also depends on various influencing variables. For example, as with text-based persuasion processes (Kapoor et al. 2020), recipients’ attributed credibility also influences image persuasiveness (Kessler et al. 2016).

Attributed credibility can generally be said to have a great influence on beliefs and thus on the effects of communication in general (Bentele 1988; Pornpitakpan 2004). One of the most important criteria used to filter information and judge it as reliable is credibility. The persuasiveness of a communication is strongly dependent on whether it is perceived as credible or not (Kapoor et al. 2020; Pornpitakpan 2004; Valentini 2018). As an important heuristic, credibility is a filter in the process of knowledge acquisition and simultaneously controls this process (Bentele 1988; Schweiger 1998). Ultimately, credibility research examines any variables that can be used to make decisions and judgements about the credibility of a piece of information (Reinhard and Sporer 2010; Valentini 2018). In situations of uncertainty, credibility provides necessary orientation. Credibility is based on the subjective perception that a piece of information corresponds to the truth and thus determines the recipient’s degree of willingness to adopt the information received from the source as cognition (Eisend 2006, Valentini 2018). Credibility can already be defined as a property attributed to people, institutions, or their communicative products (oral and written texts, audio-visual representations) by someone (recipient) in relation to something (events, facts, etc.) (Bentele, 1988, p 408; Valentini 2018). Credibility arises in communication starting from the recipient and consequently must be viewed and measured as attribution from a recipient-oriented perspective (Bentele 1988; O’Keefe 2002; Roberts 2010; Schweiger 1998). Credibility is a hypothetical construct that arises from interactions between source, message and recipient (Kapoor et al. 2020; Roberts 2010).

In the study by Kessler et al. (2016), the credibility attributed to a journalistic article with different illustrations was shown to be a mediator in the persuasion process of the article content on the attitudes of the recipients. In this specific case, this means that a high credibility assessment by the recipients influenced the attitudes. Participants who saw the articles with pictures rated the content as more credible than those who read the version without pictures and recipients who found the articles more credible also had stronger attitude changes.

However, even though images are increasingly being used in online communication, the field of visual persuasion in the debunking context is not yet well researched and the current state of research does not reveal how debunking strategies work with different evidence-based images in relation to different scientific issues and health myths (Cook 2019).

- *Research Question 2: What is the persuasive effect of different images in corrective messages about health myths?*
- *Hypothesis 2: A corrective message with a machine-technical created image has a stronger persuasive effect on recipients than the same message containing a diagram; which has stronger effect than the same message containing an image of an expert; which has a stronger effect than the same message without an image.*

## Method

We conducted an online survey experiment with a 4 (myths) × 4 (picture) mixed study design. The survey participants were recruited by the German survey institute respondi AG. The sample size of 700 individuals was calculated using a priori power analysis.^1^ Quota sampling was used according to age, gender and education with regard to the total German population aged 18–74.^2^ In the first step of the questionnaire, participants had to declare how strongly they believe (t1) in nine different and (in the German-speaking area) widespread health myths. Four of these nine myths are relevant for the experiment: “alcohol is good for digestion” (alcohol myth), “picking the nose is unhealthy” (nose myth), “finger cracking leads to rheumatism or arthritis” (finger myth) and “dim light is bad for the eyes” (eye myth). Then, up to a maximum of two corrective articles versions for the relevant myths in which the participant believed were randomly assigned. The maximum of two myths per recipient was set in order to avoid that the survey takes too long. However, if a person only believed in one of the relevant myths, he or she was only given one debunking text. If a person did not believe in any of the relevant myths, he or she did not receive a debunking text. That means based on previously given answers, participants were assigned and exposed from case to case to a varying number of debunking texts. Regarding the relevant myths that the recipients believe in, the recipients are then asked about their feelings and behaviour in relation to the myths.^3^

The corrective messages were created based on the research about debunking strategies for misinformation. As Cook and Lewandowsky (2011) recommend in their debunking handbook, the myths should not be mentioned at the very beginning of the message (the heading) of an article, the myths should not repeated frequently and before they are explicitly mentioned, there should be a warning that they are only myths. In addition, alternative/plausible scientific explanations have been inserted in the debunking texts that can close gaps in recipients’ mental models; they are formulated with low lexical complexity, contain evidence and even describe why the health myth is disseminated (see also Lewandowsky et al. 2012; Ecker et al. 2015; Swire and Ecker 2018). The debunking texts were formulated as simple, easily understandable and brief, with clear language. The online news articles contained contextual information to refute the health myth and scientific evidence (expert opinion and scientific study) that argues against the myth (Pandey et al. 2014). The scientific studies were modified such that the number of test subjects, study location and mentioned details about study conduct were the same in all articles. The form of the articles and the included images also look as identical and real as possible. The articles look like online newspaper articles and contain text and no image (control group), a diagram (which provides evidence to support debunking; see supplemental material, Fig. 1), a machine-technical created image (which fits the myth, e.g., for the finger myth an X-ray of a finger and for the eye myth that of an eye; see supplemental material, Fig. 2), or just an image of an expert (see supplemental material, Fig. 3). The respective image types were as identical as possible in size, colour, design and information contained. The texts were similarly as equal as possible regarding length (*M* = 310 ± 40 words), type of headings and subheadings, general formulations and wording, fictive author, number of arguments (3) and paragraphs (3), evidence sources (study and expert) and scientific study details (see supplemental material, Fig. 4). Table 1 shows the distribution of the sample by myth and visual experimental condition. To ensure that the recipient did not click directly away with noticing the article, the “next” button was only activated after a dwell time of 20 s. After an article was read, participants were once again questioned about beliefs, feelings and possible future behaviour (t2). Corrective effectiveness occurs when the belief, positive feelings or behavioural intention in relation to a health myth decreases. This pre−/post-measurement enables a subsequent evaluation of the possible change. For each relevant myth, the respondents were also asked the origin of their belief in it and how credible they consider each article (Roberts 2010). Each respondent was also asked about his or her level of scepticism as a personality trait and potential influence variable (Tsfati and Cappella 2005). At the very end, a debriefing was held on the objectives of the survey and the study as a whole. Table 2 describes all survey variables.

**Table 1:** Distribution of the sample by myth and visual experimental condition

*n*	no image	machine-technical image	image of an expert	diagram	total *n*
nose myth	68	74	66	67	275
alcohol myth	63	72	70	72	277
eye myth	74	74	77	75	300
finger myth	69	73	64	68	274
total *n*	274	293	277	282	1126

**Table 2:** Question categories in the online survey, its source/description, descriptive results and Cronbach’s α values

question category	source/description	descriptives; *M* (*SD*) or frequencies	Cronbach’s α
gender ^1^	female, male	*M* = 1.5 (0.50)	
education ^1^	low, intermediate, higher educational qualification	low: *n* = 239; intermediate; *n* = 224; high: *n* = 237	
age ^1^	in years	*M* = 46.9 (15.4)	
previous attitude to relevant myth (t1) ^1^	5-point scale (1 = do not agree with the statement; 5 = agree with the statement); randomised	belief, behaviour and feeling (t1; see Table 3)	
duration of article viewing	in seconds	article nose myth*: M* = 83 (80); article eye myth: *M* = 99 (135); article alcohol myth: *M* = 94 (127); article finger myth: *M* = 82 (144)	
credibility of the perceived article ^2^	5 items of the credibility scale of Roberts (2010); semantic differential; randomised; 5-point scale: (1 = not credible; 5 = credible)	article nose myth*: M* = 3.5 (0.8);	.85
		article eye myth: *M* = 3.7 (0.8);	.89
		article alcohol myth: *M* = 3.6 (0.8);	.88
		article finger myth: *M* = 3.6 (0.9)	.88
source of myth beliefs ^2^	multiple answers allowed; closed-ended question		
post attitude to relevant myth (t2) ^2^	5-point scale (1 = do not agree with the statement; 5 = agree with the statement); randomised	belief, future behaviour and feeling (t2; see Table 3) uncertainty: alcohol myth: *M* = 2.7 (1.3); nose myth: *M* = 3.0 (1.3); eye myth*: M* = 2.7 (1.2); finger myth *M* = 2.7 (1.3)	
scepticism ^2^	18 items of the scepticism scale of Hussin and Iskandar (2015); item selection: 3 items per factor with the highest factor load; 5-point scale; scale: (1 = sceptical; 5 = not sceptical)	*M* = 2.7 (0.6)	.83
survey duration ^2^	in seconds	*M* = 1013 (780)	

^1^ = questions before stimulus application; ^2^ = questions after stimulus application

All texts, images, and diagrams were pretested with 36 students in April 2019. Four pictures of experts, five diagrams, five machine-technical created images and five debunking messages were tested in an online survey. The best four of the tested debunking texts and images of the different image types were used for the study. Univariate variance analyses with repeated measures were calculated to test whether there are significant differences between the text versions and between the images of the different image types. The results of the pretest show that the expert images did not differ significantly regarding their scientific nature, authenticity, competence, attractiveness and recognisability (see supplemental material, Table 1). Further, no significant differences were found in the comprehensibility, credibility and professionalism of the diagrams and machine-technical created images. The debunking texts showed no significant differences in terms of credibility (Roberts 2010), comprehensibility, quality, emotionality and convincing power (see supplemental material, Table 1).

### Description of the sample

Seven hundred subjects (50% women) participated in the experiment. These were representative of the German population in terms of age, gender and education. Thirty-four percent had a lower level of educational qualification (Hauptschule; *n* = 236); 32% had an intermediate educational qualification (Mittel-, Real-, Handelsschule; *n* = 224) and 34% had a higher educational qualification (Abitur/university entrance qualification; *n* = 237). On average, the participants were 47 years old (*SD* = 15.4); 19% (*n* = 133) were 18–29 years old 16% (*n* = 112) were 30–39 years old 18% (*n* = 126) were 40–49 years old 21% (*n* = 147) were 50–59 years old and 26% (*n* = 182) were 60–74 years old.

## Results

The whole survey lasted an average of 17 min. The articles were viewed for a similar period: the nose myth was 83 s, the eye myth 99 s, the alcohol myth 94 s and the finger myth finger 82 s.

Paired-samples *t*-tests showed highly significant mean differences (*p <* .001) between the pairs belief t1 and t2, (future) behaviour t1 and t2 and feeling t1and t2 for all myths (Table 3). To measure the debunking effects of the different myths more precisely, we calculated repeated measures analyses of covariance (ANCOVAs) for the variables belief t1 and t2, (future) behaviour t1 and t2 and feeling t1and t2 for all myths (Table 4) with the fixed between-subjects factor of the different visual experimental conditions. Strong debunking effects can be seen for the belief in all four myths. When it comes to the influence on behaviour, there is a weak effect for the nose myth, a very weak effect for the alcohol and finger myths and a strong effect for the eye myth. Regarding the debunking effect on feelings, significant influences are shown for the nose and alcohol myths, but these are very weak. For the finger myth, the effect is weak. Regarding the eye myth, there is a medium strong debunking effect. All effects are independent of the visualisation. Overall, the strongest debunking effect can be seen for belief, with less strong effects on (future) behaviour and feelings regarding the different myths.

**Table 3:** Previous attitude and subsequent attitude about relevant health myth grouped by myth and visual experimental condition

		previous attitude about relevant health myth (t1)			subsequent attitude about relevant health myth (t2)		
		belief in myth t1; *M*	behaviour according to the myth t1; *M*	feeling according to the myth t1; *M*	belief in myth t2; *M*	future behaviour according to the myth t2; *M*	feeling according to the myth t2; *M*
	nose myth	3.70	3.12	3.00	2.76	2.41	2.67
	alcohol myth	3.45	2.28	2.63	2.51	1.97	2.35
	eye myth	3.95	3.72	3.45	2.72	2.52	2.65
	finger myth	3.62	3.07	3.22	2.56	2.64	2.62
nose myth	no image	3.65	3.22	3.13	2.76	2.41	2.81
	machine-technical image	3.58	3.04	2.86	2.84	2.32	2.55
	expert image	3.79	3.36	3.00	2.74	2.45	2.68
	diagram	3.81	2.85	2.85	2.69	2.48	2.64
alcohol myth	no image	3.51	2.38	2.78	2.43	1.87	2.33
	machine-technical image	3.38	2.08	2.42	2.43	1.82	2.14
	expert image	3.41	2.13	2.56	2.39	1.91	2.24
	diagram	3.50	2.54	2.79	2.76	2.28	2.69
eye myth	no image	3.95	3.76	3.47	2.74	2.49	2.74
	machine-technical image	3.88	3.51	3.45	2.74	2.50	2.64
	expert image	4.04	3.83	3.66	2.58	2.56	2.48
	diagram	3.92	3.77	3.23	2.81	2.52	2.76
finger myth	no image	3.67	3.16	3.26	2.68	2.74	2.80
	machine-technical image	3.59	2.84	3.00	2.42	2.53	2.45
	expert image	3.55	2.98	3.25	2.64	2.75	2.70
	diagram	3.68	3.31	3.38	2.50	2.53	2.53

5-point scale (1 = do not agree at all; 5 = agree completely)

**Table 4:** Repeated measures ANCOVAs

	variables^1^ belief t1 and t2	variables^1^ (future) behaviour t1 and t2	variables^1^ feeling t1and t2
nose myth; *n* = 275	*F*(1 271) = 169.1, *p* < .001, partial η^2^ = .384; *f* = 0.49	*F*(1 271) = 64.4, *p* < .001, partial η^2^ = .192; *f* = 0.21	*F*(1 271) = 11.3, *p* < .001, partial η^2^ = .040; *f* = 0.04
eye myth; *n* = 277	*F*(1 296) = 284.0, *p* < .001, partial η^2^ = .490; *f* = 0.69	*F*(1 269) = 205.4, *p* < .001, partial η^2^ = .410; *f* = 0.53.	*F*(1 269) = 85.5, *p* < .001, partial η^2^ = .224; *f* = 0.25
alcohol myth; *n* = 274	*F*(1 273) = 182.6, *p* < .001, partial η^2^ = .401; *f* = 0.52	*F*(1 273) = 24.8, *p* < .001, partial η^2^ = .083; *f* = 0.09.	*F*(1 273) = 19.3, *p* < .001, partial η^2^ = .066; *f* = 0.07
finger myth; *n* = 300	*F*(1 270) = 181.9, *p* < .001, partial η^2^ = .403; *f* = 0.52	*F*(1 270) = 22.9, *p* < .001, partial η^2^ = .078; *f* = 0.08.	*F*(1 270) = 53.3, *p* < .001, partial η^2^ = .165; *f* = 0.18

Requirements for the calculations of a repeated measures ANCOVA are given for all analyses; normal distribution of the dependent variables is given; the box tests for equality of the covariance matrices in the analyses are always not significant; sphericity is assumed for all analysis

^1^Between-subjects factor was the visual experimental condition and in each case not significant

Backfire effects may occur when debunking myths, as described in section 3. Thus, if the belief in a myth increased after correction, this can be considered a backfire effect. On the scale of 1 to 5, the belief in a myth mostly increased by only one scale point (Table 5). Thus, the backfire effects were rather weak. In this study, these effects were found in (only) one in 10 persons. When backfire effects were observed, it was investigated which person and stimulus variables supported them. For all myths, the beliefs t1 were shown to be relevant variables (nose myth *t*(38) = 3.9, *p <* .001; eye myth *t*(298) = 4.2, *p <* .001; alcohol myth *t*(33) = 2.6, *p <* .05; finger myth *t*(45) = 3.8, *p <* .001). However, the beliefs t1 were surprisingly less pronounced for the debunking effect cases for all myths (that is, more moderate). For the eye myth, age was also shown to be influential (*t*(298) = −2.3, *p <* .05). Older persons were more likely to be subject to a backfire effect.

**Table 5:** Backfire effects

change in belief (scale 1–5)	nose myth *n* (%)	eye myth *n* (%)	alcohol myth *n* (%)	finger myth *n* (%)
−4	5 (1.8)	14 (4.7)	3 (1.1)	8 (2.9)
−3	26 (9.5)	26 (8.7)	17 (6.1)	20 (7.3)
−2	54 (19.6)	84 (28.0)	82 (29.6)	86 (31.4)
−1	81 (29.5)	94 (31.3)	60 (21.7)	63 (23.0)
0	84 (30.5)	59 (19.7)	92 (33.2)	69 (25.2)
+1 ≙ backfire effect	22 (8.0)	18 (6.0)	20 (7.2)	20 (7.3)
+2 ≙ backfire effect	3 (1.1)	5 (1.7)	3 (1.1)	8 (2.9)

If the difference in number between belief t1 and t2 is calculated as a variable, significant correlations of the change are observed with mean strength. For all myths, the belief change correlates with the credibility attributed to the respective debunking article (nose myth *r*_Sp_ = .34, *p <* .001; eye myth *r*_Sp_ = .35, *p <* .001; alcohol myth *r*_Sp_ = .38, *p <* .001; finger myth *r*_Sp_ = .35, *p <* .001). The more credible a debunking article is judged, the greater the recipients’ intended belief change. A significant correlation with low strength of the eye (*r*_Sp_ = .19, *p <* .01), alcohol (*r*_Sp_ = .15, *p <* .05) and finger (*r*_Sp_ = .16, *p <* .01) myths was also found with the duration in which the respective debunking articles were viewed. The longer a recipient viewed a debunking article, the greater the intended belief change.

If we include all covariates in the calculated ANCOVAs, for the nose myth, the credibility of the article content (*F*(1 223) = 23.9, *p <* .001, partial η^2^ = .097; *f* = 0.09) and the scepticism of the respondents (*F*(1 223) = 8.8, *p <* .001, partial η^2^ = .038; *f* = 0.04) have a significant interaction effect on debunking but with very small effect size. The same is true for the variable credibility of the article content about the eye myth (*F*(1 253) = 24.0, *p <* .001, partial η^2^ = .087; *f* = 0.09) and the alcohol myth (*F*(1 236) = 21.8, *p <* .001, partial η^2^ = .085; *f* = 0.09). For the finger myth, the same appears for credibility of the article content (*F*(1 234) = 18.0, *p <* .001, partial η^2^ = .071; *f* = 0.07) and duration of article viewing (*F*(1 234) = 5.6, *p <* .05, partial η^2^ = .023; *f* = 0.02). Neither duration of article viewing for the finger myth nor scepticism for the nose myth show significant correlations with the output variable. Taking all myths together, it is evident in the one-way analysis of variance (ANOVA) that only the credibility of the article content is generally a significant covariate with respect to the belief in a myth at the time t2, but this has a small effect size (*F*(1 1118) = 149.9, *p <* .001, partial η^2^ = .117; *f* = 0.12). Even recipients’ scepticism had no significant influence on the corrective effectiveness of the debunking texts. As also indicated in Table 2: The respondents’ scepticism was in the middle range. There was no direct connection between scepticism and prior beliefs t1 to the relevant health myths.

Taking all myths together, it is evident that no significant correlations can be found between the credibility attributed to a debunking article’s content and the image type used or the belief in a myth at time t2; the variable credibility of the article content is independent. The more credible a debunking text is for a recipient, the more corrective effectiveness it had.

Respondents rated the credibility of the online articles as rather credible. The attributed credibility did not differ significantly in terms of the different image types for all myths. The articles were judged to be equally rather credible (see Table 2). Mediation analyses according to Hayes (2017) were calculated for each health myth. For all four health myths, there was no significant mediation of credibility in the effect of the picture types on the change in beliefs of the recipients. Only the direct effect of attributed credibility on belief change was significant. All other direct and indirect effects were not significant. Attributed credibility does influence the effectiveness of debunking, but image types do not significantly influence the attributed credibility and belief change. Overall, there is evidence to support hypothesis 1: the more credible a debunking text is for a recipient, the more corrective effectiveness it had.

By means of *t*-tests with independent samples, we calculated for each myth whether the image type has an influence on belief t2, future behaviour t2, or feeling t2. There were no significant differences between the different image types. Even if the image type is defined as a dummy variable (with image vs. without image), no significant differences were found. Thus, the results confirm the findings of the ANCOVAs with repeated measurement that the image type was not a decisive factor in this investigation. The texts worked well as debunking tools with and without images. Hypothesis 2 must be rejected: The corrective messages do not differ in their persuasive effects depending on the image types used.

## Discussion

In times of fake news and conspiracy theories, which are rampant on the internet, it is important for a well-informed society to strengthen social trust in facts and science in general. By identifying debunking strategies for mis- and disinformation, we can disseminate correct (health) information, prevent the spread of more misinformation and help people to better understand why and when they can trust scientific advice. In addition, knowing debunking practices specifically related to health issues will, at best, result in less money spent on dubious medical treatments and more confidence in effective, evidence-based treatments (Betsch and Sachse 2013). The results also contribute to the still relatively unexplored field of research on visual persuasion (Pandey et al. 2014), especially in the debunking context and the context of pre-existing beliefs.

The results of this study can show, among other things, that receiving an online news article that refutes a widespread health myth with or without an image can significantly change the recipient’s beliefs toward this myth. This finding is consistent with meta-analyses by Blank and Launay (2014), Walter and Murphy (2018) and Walter and Tukachinsky (2019), which showed that corrective messages can significantly reduce belief in misinformation and therefore presenting them is usually advantageous if the message is appropriately designed. The debunking articles were designed according to the correction recommendations of Cook and Lewandowsky (2011), Ecker et al. (2015), Lewandowsky et al. (2012); Pandey et al. (2014) and Swire and Ecker (2018). The debunking went very well, despite the poor conditions: correction of real-world misinformation is challenging, the myths were mostly attributed to a credible source, the misinformation was repeated multiple times prior to correction and there was a time lag between the delivery of the myth and the correction (Walter and Murphy 2018; Walter and Tukachinsky 2019).

Our study did not show an increased persuasion power through images. This result does not correspond with the assumptions and investigations of, for example, Holicki (1993), Houts et al. (2006) and Nyhan und Reifler (2019). Further, the results did not provide scientific evidence for the assumption that the persuasion power of the different image types differs in the correction of health myths. This contradicts the findings of McCabe and Castel (2008) and Kessler et al. (2016). Research on misinformation shows that illustrated articles with misinformation are considered as more credible than text-only stimuli (Dan 2021; Smelter and Calvillo 2020). We were unable to demonstrate this for debunking texts in this study. One possible explanation: the debunking texts were so well and unambiguously formulated that the evidence power of the images had no further influence on the attitude changes. Perhaps the debunking texts themselves were so effective, because they are based on the latest research findings, that the recipients simply did not care what picture was shown. In contrast to previous studies that found significant differences, in this investigation, the article content itself was not as controversial as in Kessler et al. (2016) and no first-time information was conveyed about a topic on which the recipient had no opinion, as in McCabe and Castel (2008). Further research on when images generate evidence and persuasively affect settings is needed here.

The extent to which the correction articles worked with or without an image also depended on the article’s attributed credibility. There was a significant influence of credibility on belief change, with a higher credibility based on a stronger belief change. The actual potential for effect arose through the complex interrelation between the stimulus and the recipient variables. The attributed credibility of a message was a decisive influencing variable (as suspected, according to O’Keefe 2002; Pornpitakpan 2004). For scepticism, no influence on belief change could be found, which contradicts the results and assumptions of DiFonzo et al. (2016), Lewandowsky et al. (2012) and Swire and Ecker, 2018.

The backfire effects found in this study can be justified by neither a stronger prior belief nor other personal and stimulus characteristics, which contradicts the assumptions of Lodge and Taber (2000), Hart and Nisbet (2012) and Nyhan and Reifler (2014). Thus, these are presumably not worldview backfire effects. Since the selected health myths are so fluent and familiar, it can be assumed that they result from familiarity backfire effects (Berinsky 2017; Cook and Lewandowsky 2011; Lewandowsky et al. 2012). There are some studies showing that the backfire effect increases over time (e.g., Peter and Koch 2016). Future studies should consider such long-term effects.

The interpretation of the results and the associated validity of the experiment are subject to certain methodological limitations. Thus, the experiment may suffer from reactivity and lack of standardisability due to its artificial interview situation. Interference variables, such as the test subjects’ attention and distraction, are difficult to control in web surveys. In addition, participants may respond in a socially desirable way or try to present themselves in a good light because of the transparent query about their conscious, verbalisable attitudes. The debunking stimuli were artificially created, which may limit their external validity and generalisability. Further, we do not know whether there might be different persuasive effects of the image types for less good created debunking texts. We cannot make any statements on which precise point in the debunking texts promotes the effective debunking, either. Even with the minimum dwell time, we do not know whether the recipients attentively read the articles and images or observed the pictures. Here, eye tracking studies could generate deeper insight and be used to check how long and in what detail the text and image of the correction message are viewed and whether the viewing time differs between the different image types. Future research should also carry out a further attitude measurement after a certain period and not only directly measure the attitudes after article reception, to be able to (better) measure the continuous influencing effect (Walter and Tukachinsky 2019).

Despite these limitations, the results of this study offer an optimistic outlook on the correction of health-related misinformation and especially health myths. In the current era, with extensive discussion about a post-truth society and fake news, this work can offer insight into why and how people change their beliefs (or not) and how beliefs in health myths can be reduced. While it is imperative to address the influence of misinformation, research also finds that poorly designed interventions can be ineffective or counterproductive (Cook 2019). The understanding of how different evidence representations and images work and which factors and processes influence (visual) persuasion and the refutation of misinformation can be used by journalists, scientists, doctors and many other actors for efficient (online) communication. Thus, the findings can be applied not only in the field of health communication but to effectively counteract misinformation, myths, or fake news on the Internet in general to avoid potential negative social or individual effects as much as possible.

## Supplementary Information

ESM 1 (DOCX 5848 kb)

## Data Availability

The study material (stimuli) is supplied in the supplemental material to the study. The authors confrm that all relevant data are included in the article.

## Footnotes

1 At least 619 subjects are necessary after calculation by G-Power (www.gpower.hhu.de/) to identify small to medium effects (f = .20) between two groups (e.g., variables belief t1 and t2) in an analysis of covariance (ANCOVA with max. α-error = .05 and power (1-ß-error) = .95) with multiple covariates.

2 Age: five steps: 16% of 18–29-year-olds; 18% of 30–39-year-olds; 21% of 40–49-year-olds; 19% of 50–59-year-olds; and 26% of 60–74-year-olds. Education: three steps: 34% with low, 32% with intermediate, and 34% with higher educational qualification. Gender: 50% women. The quotation was made by the survey institute and is based on the current data of the German Federal Statistical Office.

3 Example myth alcohol: behaviour: “I often drink a glass of alcohol when I feel full because it is good for digestion” and feelings “I feel better when I feel full because it is good for digestion”.

## Code availability

Code available on request from the corresponding author.
