# Supplementary material for: Debunking health myths on the internet: the persuasive effect of (visual) online communication
Source: Z Gesundh Wiss. 2022 Jan 19;30(8):1823–35. doi: 10.1007/s10389-022-01694-3 (PMC8766354; doi:10.1007/s10389-022-01694-3)
Supplement: Supplementary file 1 — (DOCX 5848 kb) [file 10389_2022_1694_MOESM1_ESM.docx]

**Supplemental Material**

Table 1: Evaluation of the different images and debunking messages

|  |  | comprehensibility *M(SD)* | credibility^1^ *M(SD)* | professionalism *M(SD)* |  |  |
| --- | --- | --- | --- | --- | --- | --- |
| diagram | alcohol | 4.36 (.72) | 3.71 (.85) | 3.86 (.99) |  |  |
|  | nose | 4.33 (.86) | 3.78 (.78) | 4.11 (.67) |  |  |
|  | eye | 4.50 (.85) | 3.76 (.74) | 3.64 (1.10) |  |  |
|  | finger | 4.28 (.94) | 3.47 (1.10) | 3.61 (1.08) |  |  |
| machine-technical image | alcohol | 3.56 (1.05) | 3.76 (.65) | 4.14 (.68) |  |  |
|  | nose | 3.61 (1.18) | 3.88 (.61) | 4.44 (.65) |  |  |
|  | eye | 3.56 (1.13) | 3.84 (.61) | 4.47 (.74) |  |  |
|  | finger | 3.50 (1.11) | 4.04 (.55) | 4.50 (.70) |  |  |
|  |  | comprehensibility *M(SD)* | credibility^1^ *M(SD)* | quality *M(SD)* | emotionality *M(SD)* | convincing power *M(SD)* |
| text | alcohol | 4.75 (.44) | 3.93 (.67) | 3.83 (1.00) | 2.03 (1.13) | 4.36 (.72) |
|  | nose | 4.75 (.55) | 3.96 (.66) | 3.89 (.98) | 1.86 (.99) | 4.39 (.76) |
|  | eye | 4.75 (.50) | 4.13 (.64) | 3.78 (.90) | 1.94 (.98) | 4.58 (.55) |
|  | finger | 4.72 (.51) | 3.92 (.80) | 3.89 (1.04) | 2.14 (1.15) | 4.33 (.86) |
|  |  | Scientific nature *M(SD)* | authenticity *M(SD)* | attractiveness *M(SD)* | competence *M(SD)* | recognisability *M(SD)* |
| expert | 1 | 3.61 (1.46) | 4.42 (.77) | 3.67 (.83) | 4.44 (.70) | 4.75 (.44) |
|  | 2 | 3.53 (1.16) | 4.00 (.96) | 3.14 (.80) | 4.17 (.88) | 4.47 (.77) |
|  | 3 | 3.69 (1.17) | 3.97 (1.06) | 3.53 (.77) | 4.19 (.79) | 4.28 (.97) |
|  | 4 | 3.47 (1.21) | 3.81 (1.04) | 3.78 (.83) | 4.11 (.92) | 4.00 (1.35) |

*Note: n = 36, All characteristics were considered with one question each except credibility. ^1^ Cronbach’s alpha for credibility scale (Roberts, 2010): machine-technical image = .75; diagram = .92*

Figure 1: Diagrams used in the four different debunking messages (in German)

**
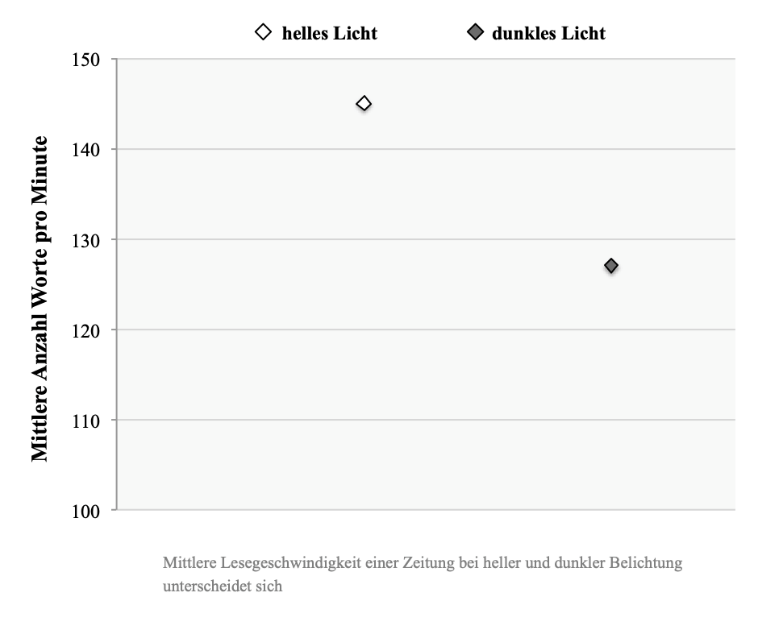

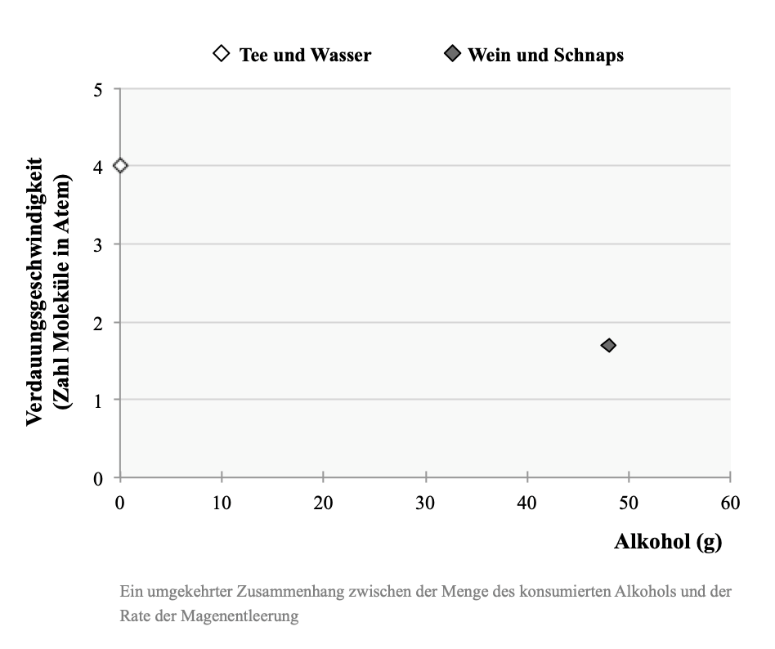
**

**
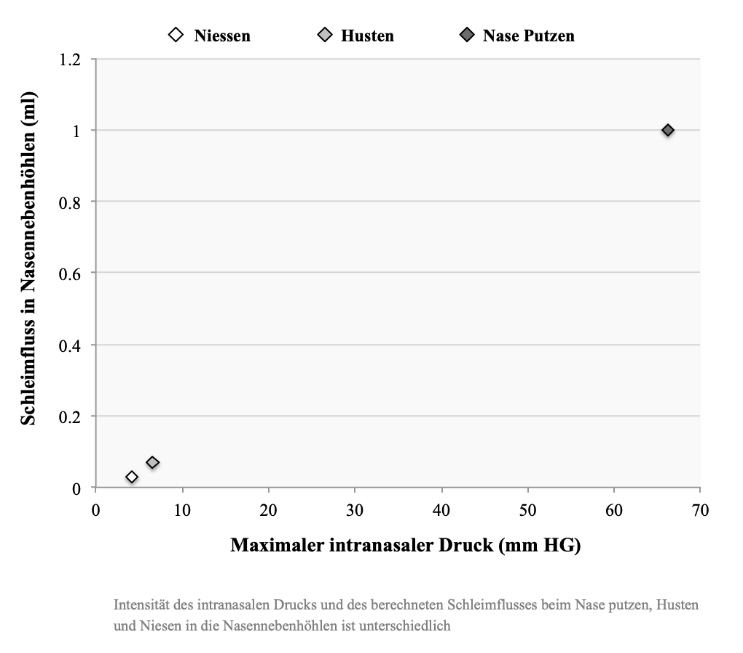
**

**
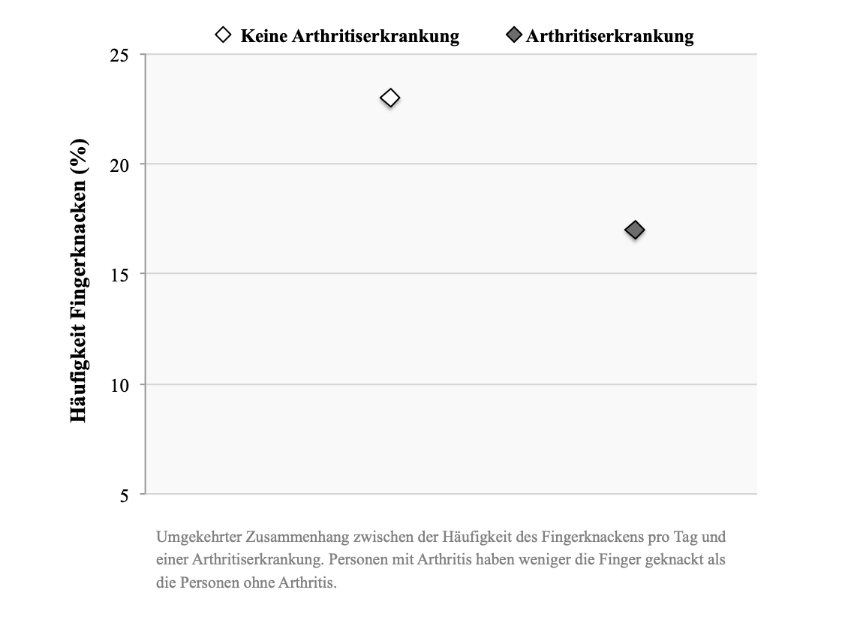
**

Figure 2: The machine-technical created images used in the four different debunking messages

**
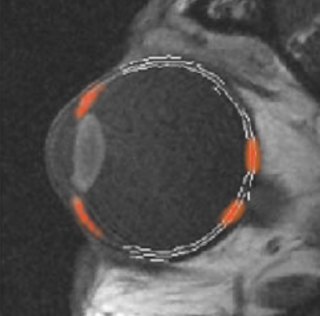

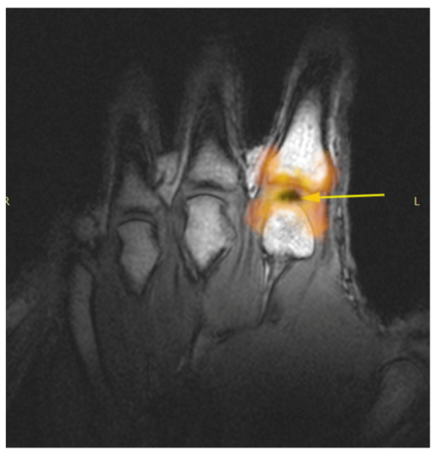

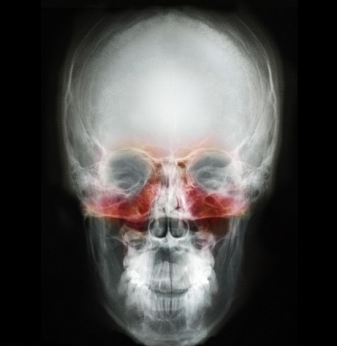

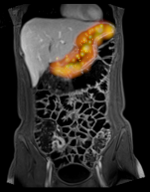
**

Figure 3: Expert images used in the four different debunking messages

**
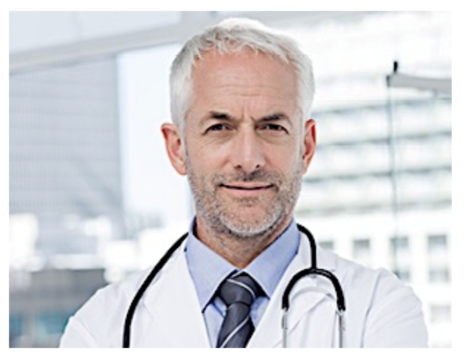

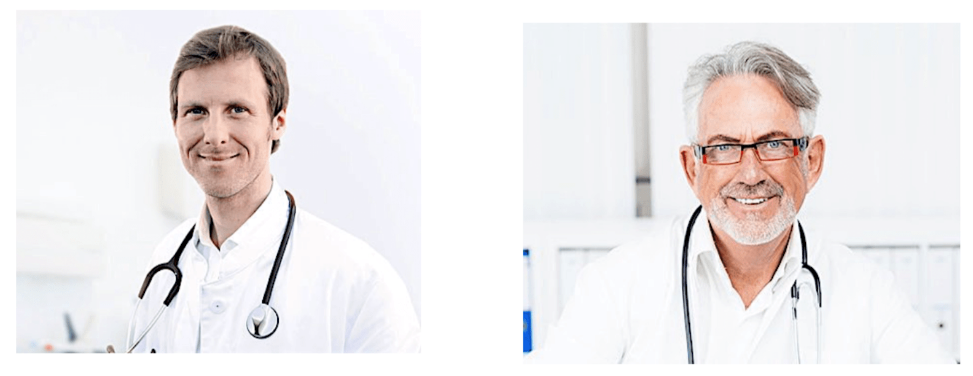

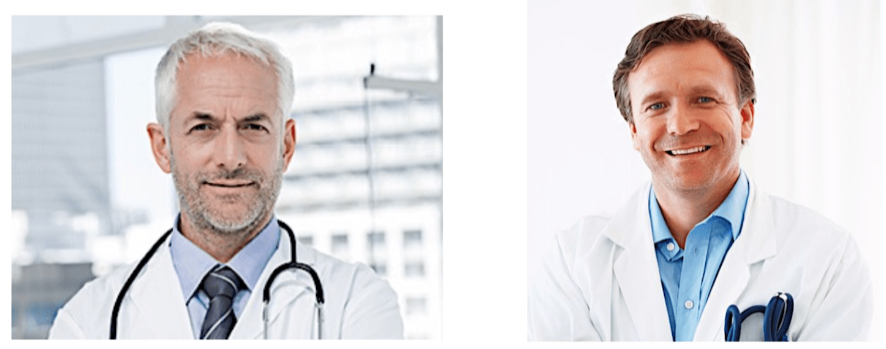
**

Figure 4: The four final debunking texts with the corresponding images in German (original) and English

**
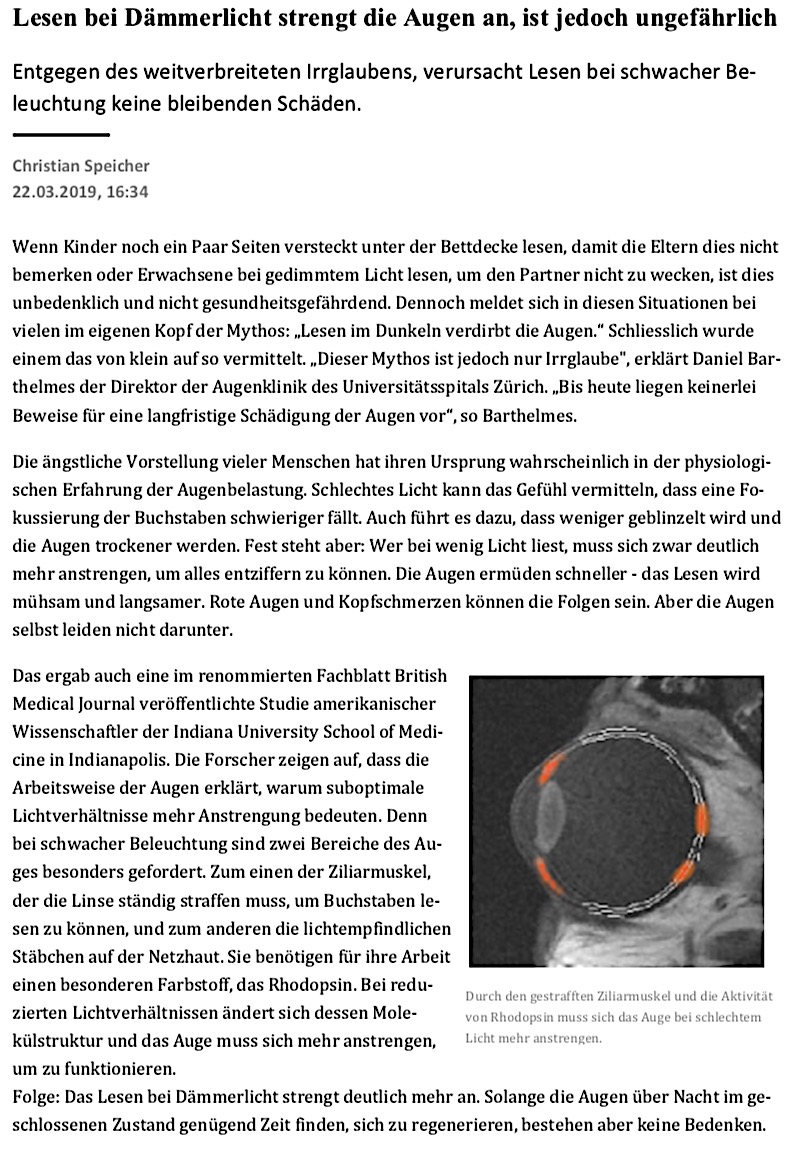

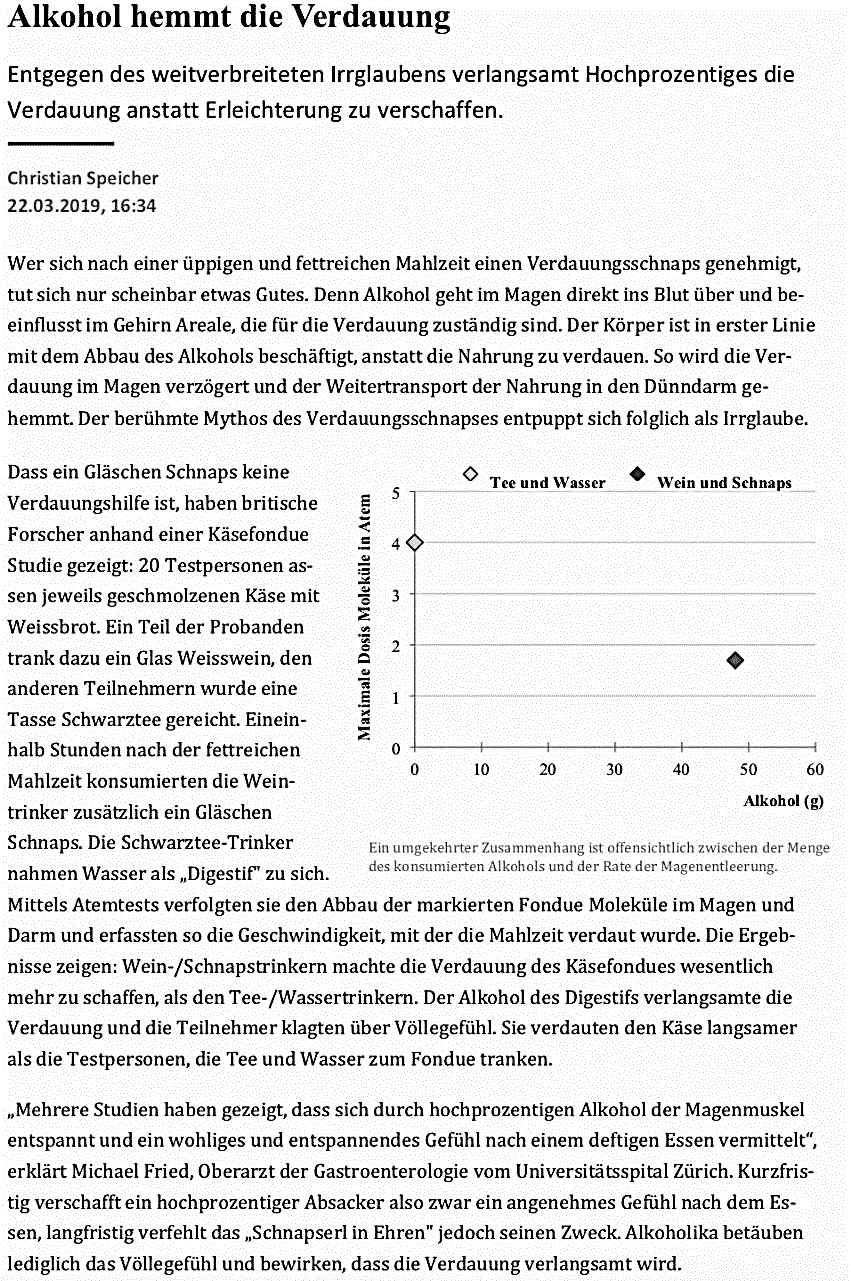
**

**
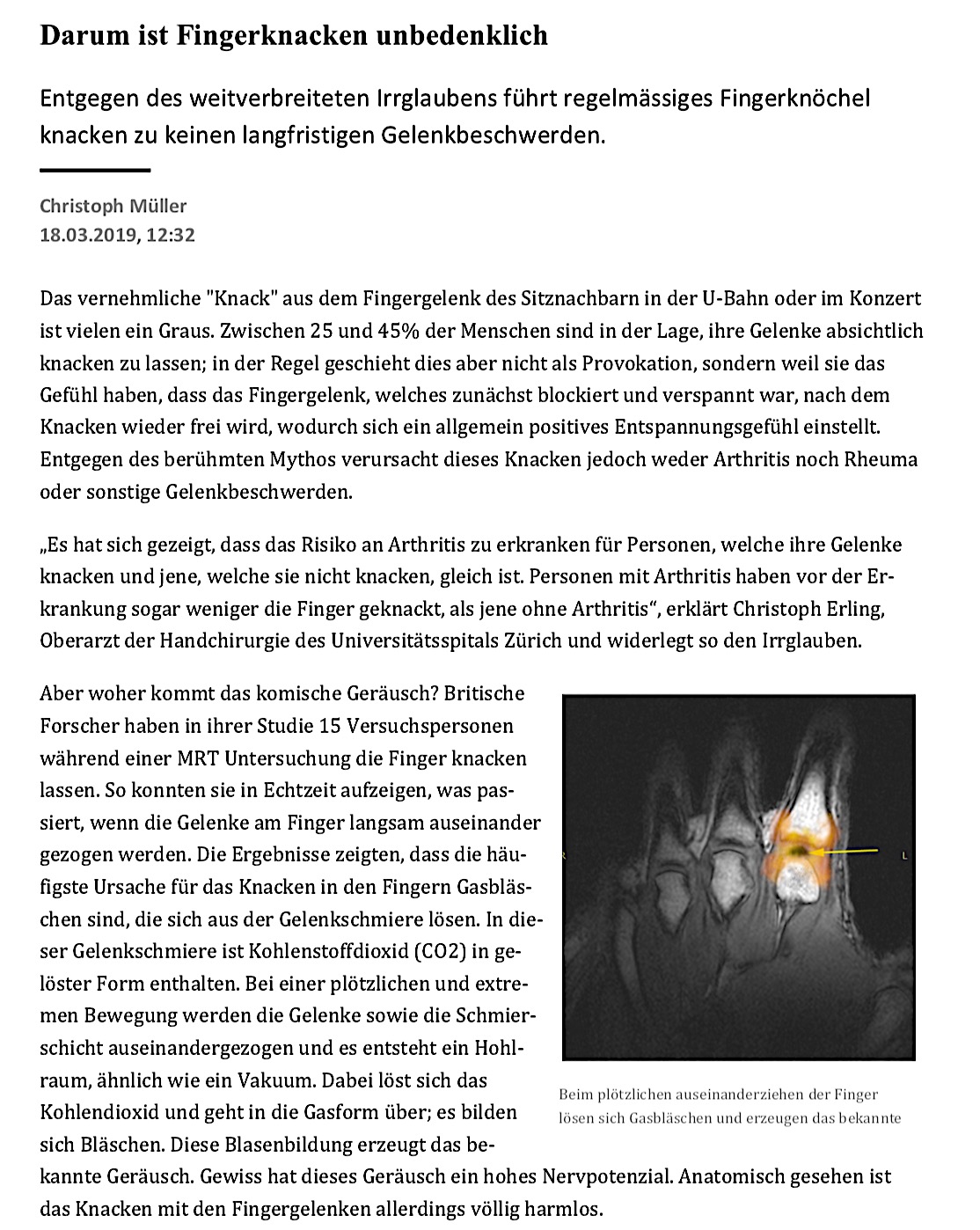

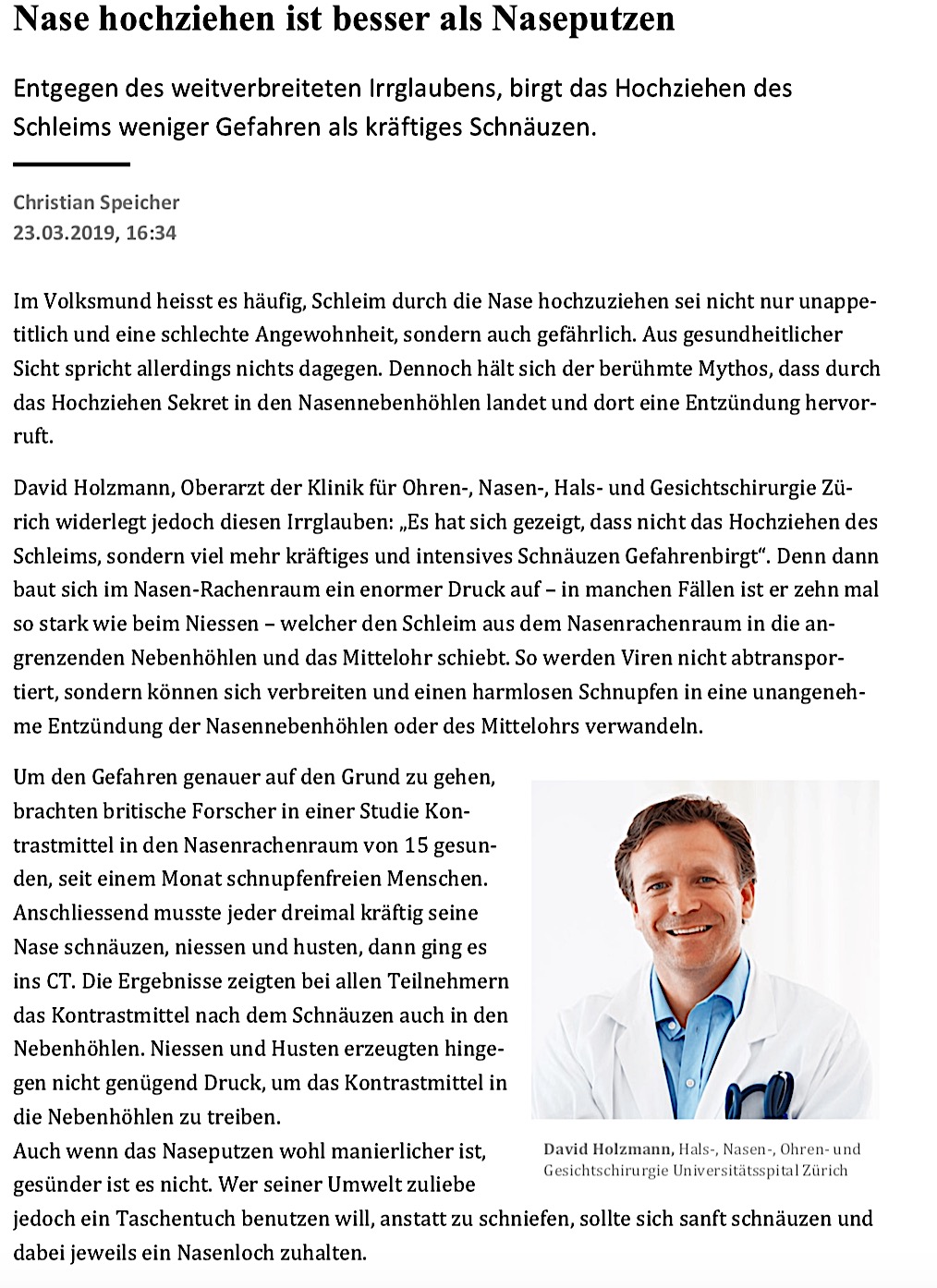
**

**Alcohol inhibits digestion**

Contrary to the widespread misconception, liquor slows down digestion instead of providing relief

Those who allow themselves a digestive liquor after a sumptuous and greasy meal are assumed to be doing themselves some good, but alcohol passes directly into the bloodstream in the stomach and influences areas of the brain that are responsible for digestion. So the body is primarily dealing with degrading alcohol instead of digesting food. This delays digestion in the stomach and inhibits transport of food into the small intestine. Therefore, the famous myth of the digestive liquor turns out to be a misconception.

British researchers have shown in a cheese fondue study that a glass of liquor is no aid to digestion: 20 participants ate melted cheese with white bread. Some of the participants drank a glass of white wine, and the others had a cup of black tea. One and a half hours after the high-fat meal, the wine drinkers also consumed a glass of liquor. The black tea drinkers consumed water as a "digestif." Using breath tests, researchers followed the degradation of the marked fondue molecules in the stomach and intestines and recorded the speed at which the meal was digested. The results show that the wine and liquor drinkers had much more trouble digesting the cheese fondue than the tea and water drinkers did. The alcohol slowed digestion, and the participants complained about feeling full. They digested the cheese more slowly than the participants who drank tea and water with the fondue.

**Wine and Liquor**

**Tea and Water**

"Several studies have shown that alcohol relaxes the stomach muscle and gives a pleasant and relaxing feeling after a heavy meal," explains Michael Fried, doctor in gastroenterology at Zurich University Hospital. In the short term, a glass of liquor may give you a pleasant feeling after a meal, but in the long term, it fails in its purpose. Alcohol only numbs the feeling of fullness and slows down digestion.

A reverse correlation is apparent between the amount of alcohol consumed and the rate of gastric emptying.

**Picking the nose is better than blowing it**

Contrary to the widespread misconception, picking the nose is less dangerous than strong blowing.

It is often said that picking the nose is not only disgusting and a bad habit but also dangerous. From a health point of view, however, there is no argument against it. Nevertheless, the famous myth persists that this causes secretions to end up in the paranasal sinuses and causes inflammation.

David Holzmann, doctor at the Clinic for Ear, Nose, Throat, and Facial Surgery at the University Hospital of Zurich, however, refutes this misconception: "It has been shown that it is not nose picking that poses a danger but rather a strong and intense nose blowing. Because then an enormous pressure builds up in the nasopharynx—in some cases, it is much stronger than when sneezing—which pushes the mucus from the nasopharynx into the adjacent sinuses and the middle ear. Thus, viruses are not transported out but can spread and turn a harmless cold into an unpleasant inflammation of the paranasal sinuses or the middle ear.”


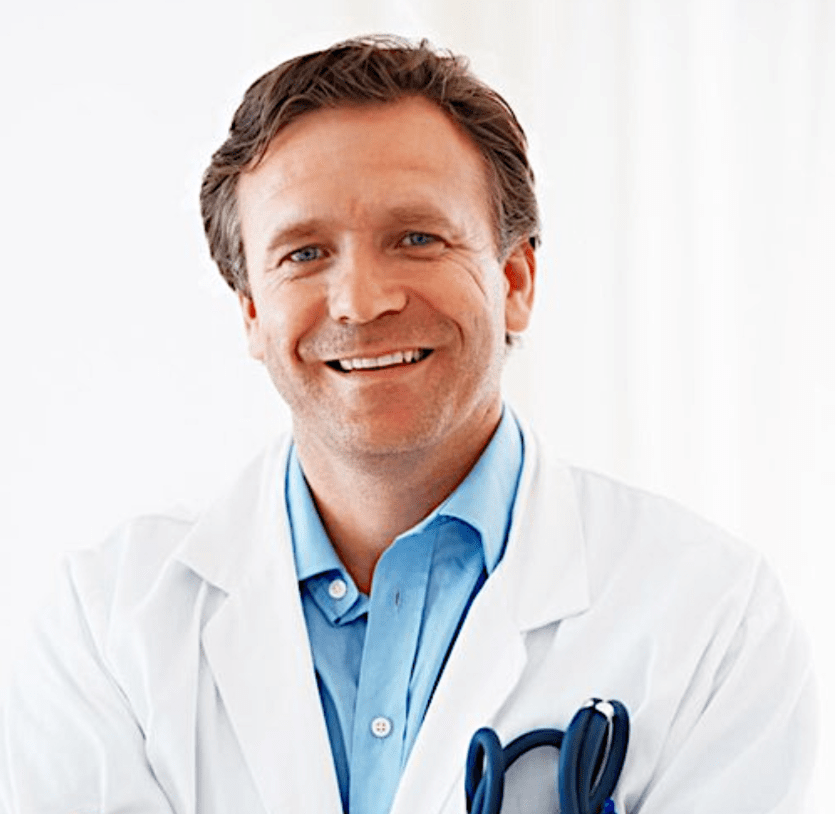
To understand the dangers in more detail, British researchers conducted a study in which contrast substances were introduced into the nasopharynx for 15 healthy participants who had been free of a runny nose for a month. Afterward, everyone had to blow the nose three times, sneeze and cough, and receive a CT scan. The results showed that the contrast substance in all participants was also present in the sinuses after nose blowing. Sneezing and coughing, on the other hand, did not produce enough pressure to drive the contrast substance into the sinuses. Even though nose blowing is probably more mannerly, it is not healthier. However, if you want to avoid nose picking, you should blow your nose gently and keep one nostril closed at a time.

**David Holzmann,** Doctor Ear, Nose, Throat and Facial Surgery, University Hospital Zurich

**That's why finger cracking is harmless**

Contrary to the widespread misconception, regular cracking of the knuckles does not lead to long-term joint problems.

The audible "crack" from the finger joint of the person sitting next to you in the subway or in concert is a nightmare for many. Between 25 and 45% of people are able to deliberately crack their joints; however, this is usually not done to provoke but because they have the feeling that the finger joint, which was initially blocked and tense, loosens up after cracking, resulting in a generally positive feeling of relaxation. Contrary to the famous myth, this cracking does not cause arthritis, rheumatism, or other joint problems.

"It has been shown that the risk of developing arthritis is the same for people who crack their joints and those who do not. People with arthritis even cracked fewer fingers before the disease than those without arthritis," explains Christoph Erling, doctor of Hand Surgery at the University Hospital of Zurich, thus refuting the misconception.


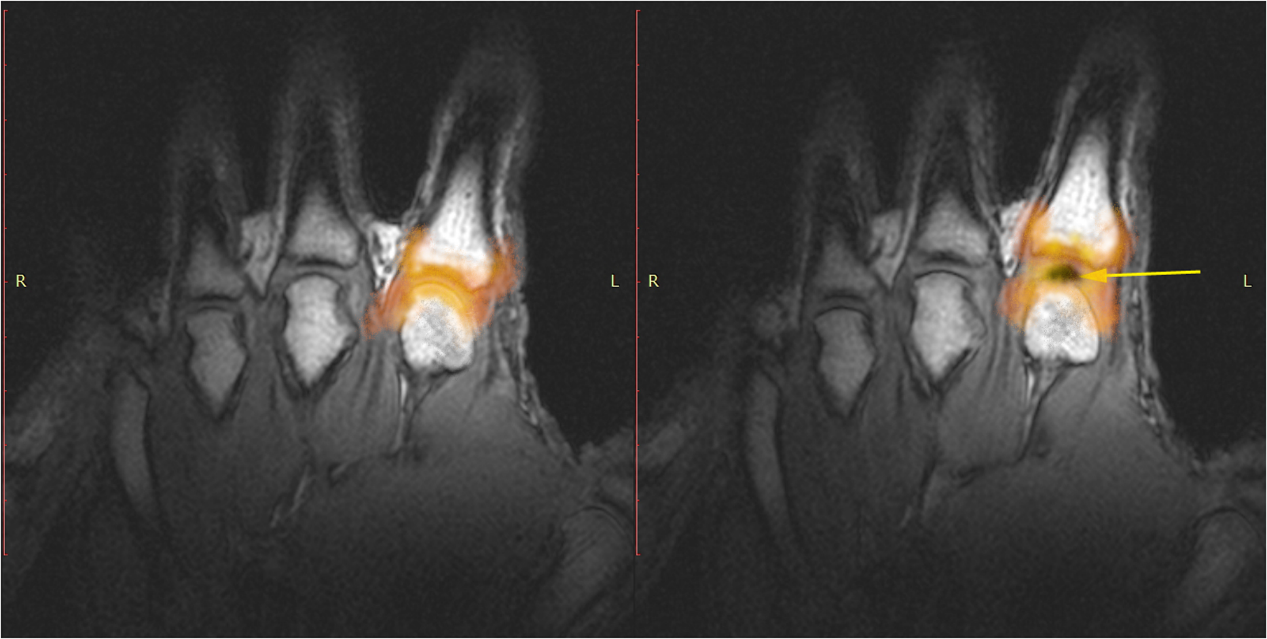
But where's that weird noise coming from? In their study, British researchers let 15 participants crack their fingers during an MRI examination. This enabled them to show in real time what happens when the joints on the finger are slowly pulled apart. The results showed that the most common cause of the cracking in the fingers is gas bubbles that are released from the synovial fluid. This synovial fluid contains carbon dioxide (CO_2_) in dissolved form. During a sudden and extreme movement, the joints and the lubricating layer are pulled apart and a hollow space is created, similar to a vacuum. The carbon dioxide dissolves and transforms into gas; bubbles form, generating the noise. This noise can certainly be annoying. Anatomically speaking, however, cracking the finger joints is completely harmless.

When the fingers are suddenly pulled apart, gas bubbles are released and produce the well-known cracking noise.

**Reading in dim light tires the eyes but is harmless**

Contrary to the widespread misconception, reading in poor lighting does not cause permanent damage.

If children still read a few pages hidden under the blanket so that their parents do not notice or adults read in dim light so as not to wake their partners, this is harmless and does not endanger their health. Nevertheless, in these situations, many people are aware of the famous myth: "reading in the dark ruins the eyes." After all, that's what you were taught from an early age. "But this myth is a misconception," explains Daniel Barthelmes, doctor at the eye clinic of the University Hospital Zurich. "Several studies have shown that no long-term damage to the eyes has occurred to date."

This frightening idea imagined by many people probably has its origin in the physiological experience of eyestrain. Poor light can make people feel that it is more difficult to focus on the letters. It also leads to less blinking and drier eyes. But one thing is certain: if you read in dim light, you will have to make a lot more effort to decipher everything. The eyes get tired more quickly—reading becomes difficult and slower. Red eyes and headaches can be the consequences, but the eyes themselves do not suffer from it.


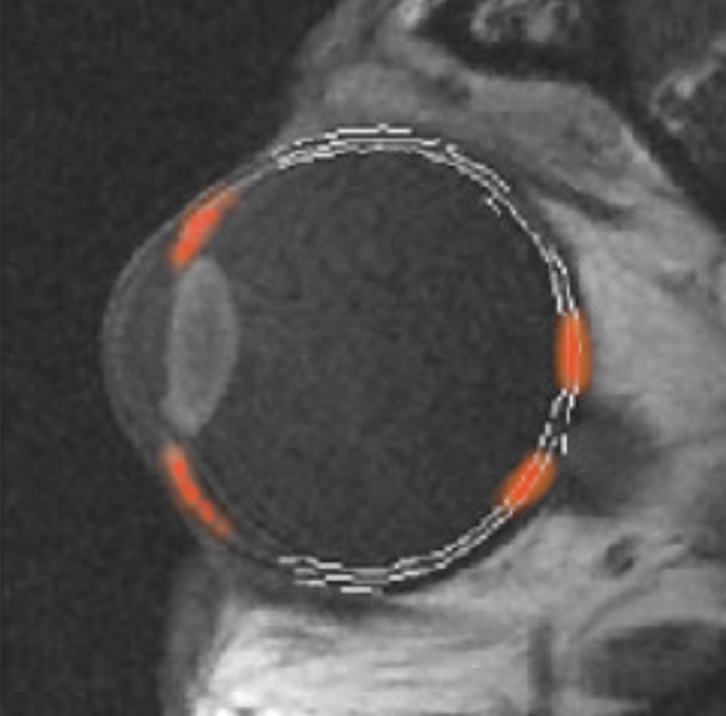
This was also the result of a study by British researchers. The 25 participants had an average reading speed of 145 words per minute under bright lighting and only 127 words per minute under dim lighting. However, the results also show that the way eyes work explains why suboptimal lighting conditions lead to more effort and slower reading. This is because two areas of the eye are particularly required under poor lighting conditions. One is the ciliary muscle, which must constantly tighten the lens to read letters, and the other is the light-sensitive rods on the retina. For their work, they need a special coloring agent, rhodopsin. When light conditions are reduced, its molecular structure changes, and the eye has to work harder to function. As a result, reading in dim light is much more tiring. However, as long as the eyes find enough time to regenerate overnight when closed, there are no concerns.

Due to the tightened ciliary muscle and the activity of rhodopsin, the eye has to work harder in poor lighting.

xt hin
